# Successful treatment of peritoneal recurrence after gastric cancer surgery with intravenous and intraperitoneal paclitaxel combined with S-1 chemotherapy: a case report

**DOI:** 10.1186/s40792-020-00849-2

**Published:** 2020-05-01

**Authors:** Kozo Miyatani, Wataru Miyauchi, Yusuke Kono, Yuji Shishido, Joji Watanabe, Takehiko Hanaki, Kyoichi Kihara, Tomoyuki Matsunaga, Manabu Yamamoto, Yoji Fukumoto, Naruo Tokuyasu, Shuichi Takano, Teruhisa Sakamoto, Soichiro Honjo, Toshimichi Hasegawa, Yoshiyuki Fujiwara

**Affiliations:** Division of Gastrointestinal and Pediatric Surgery, Department of Surgery, School of Medicine, Tottori University Faculty of Medicine, 36-1 Nishi-cho, Yonago, Tottori, 683-8504 Japan

**Keywords:** Gastric cancer, Peritoneal metastasis, Peritoneal recurrence, Metachronous peritoneal metastasis, Intraperitoneal chemotherapy, Paclitaxel, S-1

## Abstract

**Background:**

Despite recent advances in systemic chemotherapy, the prognosis of patients with peritoneal metastases from gastric cancer still remains poor. Nonetheless, several efficacious intraperitoneal chemotherapy regimens have recently been developed for patients with peritoneal metastases. However, no study has investigated the effectiveness of intraperitoneal chemotherapy for metachronous peritoneal metastases from gastric cancer after curative surgery.

**Case presentation:**

We herein report a case of a 65-year-old man who had metachronous peritoneal metastases from gastric cancer after curative total gastrectomy who had been successfully treated with intraperitoneal chemotherapy. One month after surgery, adjuvant chemotherapy with S-1 was initiated given a final pathological stage of IIIB (pT4aN2M0). However, during adjuvant chemotherapy 12 months after surgery, tumor marker levels, which had been within normal range before surgery, increased with abdominal contrast-enhanced computed tomography (CT) revealing pelvic ascites. Thereafter, staging laparoscopy was performed, and the patient was diagnosed with peritoneal recurrence of gastric cancer. Following staging laparoscopy, an intraperitoneal access port was subcutaneously implanted for subsequent intraperitoneal chemotherapy. Combined chemotherapy with intraperitoneal and intravenous administration of paclitaxel and oral S-1 was then provided. After one course of combined chemotherapy, peritoneal lavage cytology was negative for malignancy. CT showed gradually decreasing ascites, whereas tumor marker levels returned to normal. The patient continued chemotherapy without major side effects and remained progression-free for 33 months with 36 chemotherapy cycles.

**Conclusions:**

A combination regimen including intraperitoneal chemotherapy could be a promising option for patients with peritoneal recurrence after gastric cancer surgery.

## Background

Peritoneal metastasis (PM) has been found to have a significant negative impact on the prognosis of patients with advanced gastric cancer [1]. However, despite recent advances in systemic chemotherapy, the prognosis of patients with PM has still remained poor. Recently, several clinical trials, including PHOENIX-GC, have demonstrated that intraperitoneal (IP) chemotherapy was safe and promising for gastric cancer with PM [2]. However, no report has yet investigated the effectiveness of IP chemotherapy for metachronous PM after curative surgery for gastric cancer. Here, we present a case involving gastric cancer with peritoneal recurrence (PR) after curative surgery who had been successfully treated with IP and intravenous (IV) paclitaxel (PTX) combined with S-1 chemotherapy.

## Case presentation

**Fig. 1:**
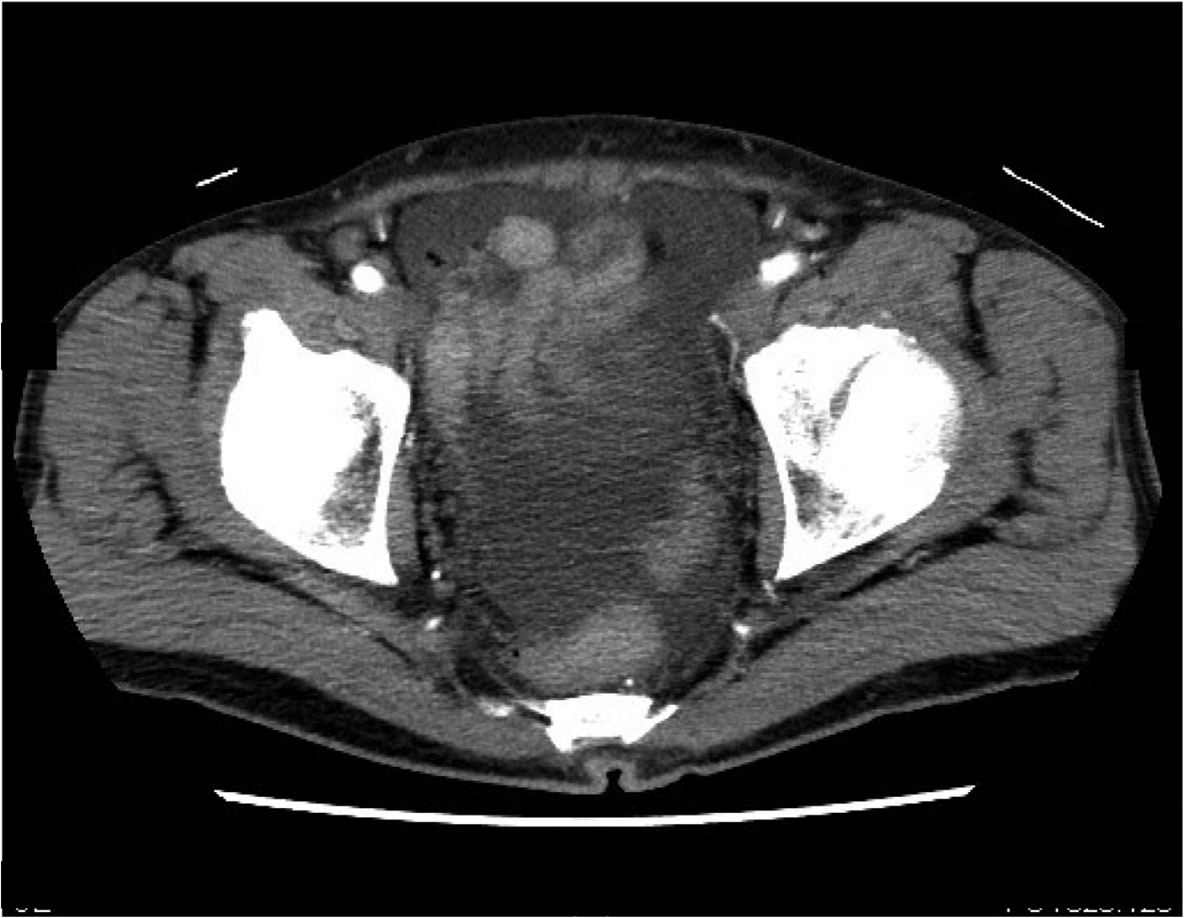
Contrast-enhanced computed tomography image before staging laparoscopy showing pelvic ascites

A 65-year-old man was referred to our department for further examination and treatment for suspected PR after gastric cancer surgery at another hospital. The patient had undergone total gastrectomy with D2 lymphadenectomy and Roux-en-Y reconstruction for gastric cancer 13 months prior. One month after the surgery, adjuvant chemotherapy with S-1 was initiated given a final pathological stage of IIIB (pT4aN2M0) according to the Japanese Classification of Gastric Carcinoma, 3rd English edition [3]. However, during adjuvant chemotherapy 12 months after his surgery, the serum levels of carcinoembryonic antigen (CEA) and carbohydrate antigen 19-9 (CA19-9), which had been within normal range before surgery, increased to 6.4 ng/mL and 44 U/mL, respectively. Despite being asymptomatic, the patient exhibited pelvic ascites on abdominal contrast-enhanced computed tomography (CT) (Fig. 1), which suggested PR.

**Fig. 2:**
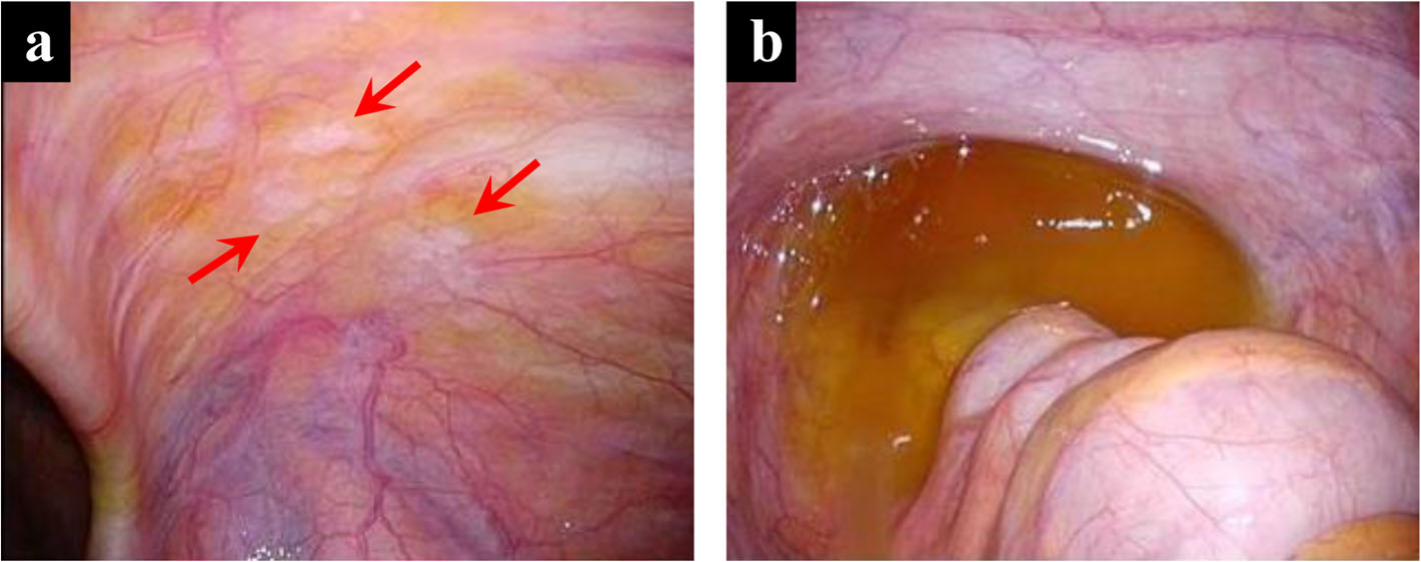
Staging laparoscopy images. **a** Disseminated white nodules on the right lower abdominal wall (arrow). **b** Moderate ascites in the rectovesical pouch

Staging laparoscopy (SL) was performed during which white nodules on the right lower abdominal wall (Fig. 2a) and ascites in the pelvic cavity (Fig. 2b) were observed. Histopathological examination of nodules revealed PM, whereas cytological examination of ascites revealed positive results for cancer cells. Accordingly, the patients were diagnosed with PR of gastric cancer with a peritoneal cancer index score of 2 [4]. During SL, an IP access port was subcutaneously implanted for subsequent IP chemotherapy. The patient was then started on combined chemotherapy with IP and IV administration of PTX and oral S-1 2 weeks after SL. Accordingly, 20 mg/m^2^ of IP PTX and 50 mg/m^2^ of IV PTX were administered on days 1 and 8, whereas 80 mg/m^2^ of S-1 was provided daily for days 1 to 14 in a 3-week cycle. PTX was diluted in 500 mL of normal saline and was administered intraperitoneally via the implanted IP access port over 1 h concurrent with an IV infusion of PTX after IP administration of 500 mL of normal saline. The chemotherapy regimen was based on the PHOENIX-GC Trial [2], a clinical study approved by the Institutional Review Board of Tottori University (approval number: C1704B011). Written informed consent was obtained from the patient included in this study.

**Fig. 3:**
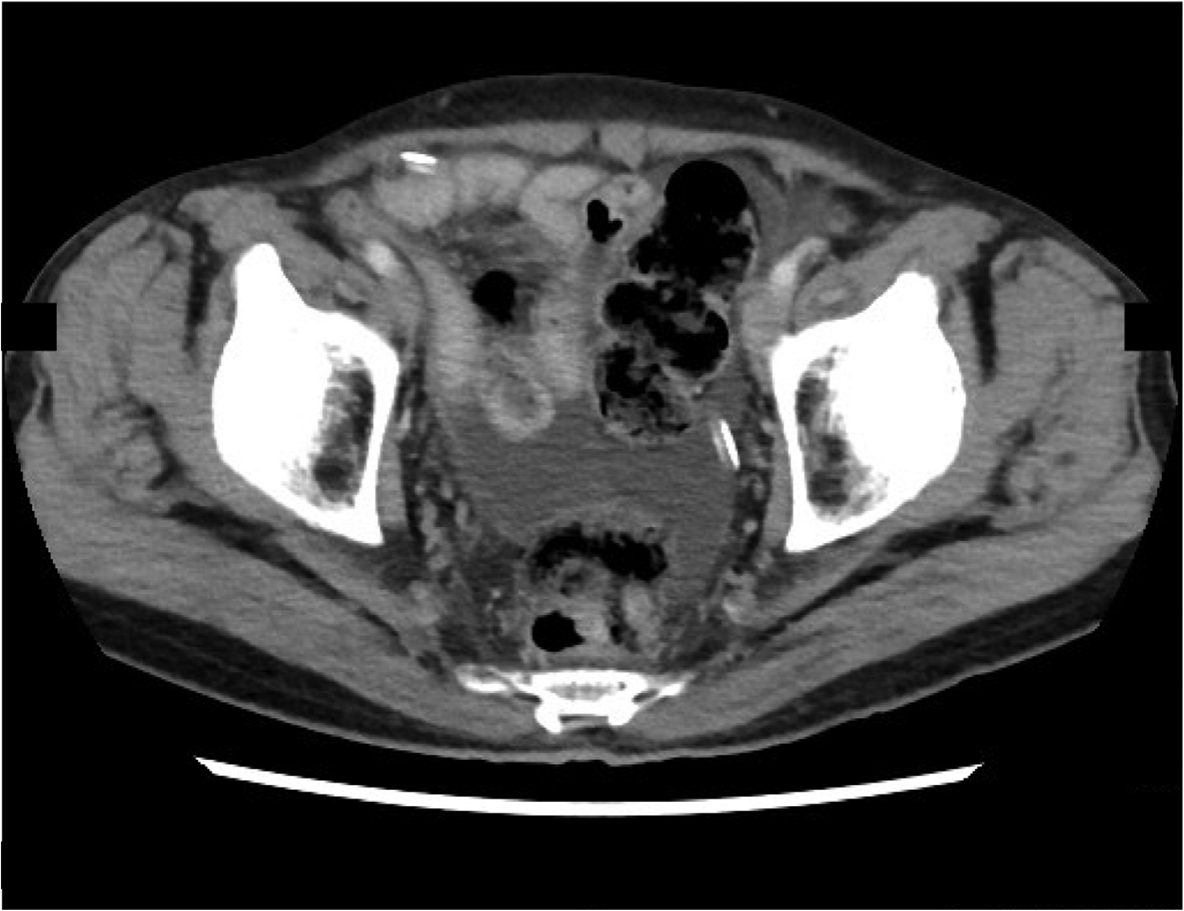
Contrast-enhanced computed tomography image after combination chemotherapy showing decreased ascites in the pelvic cavity

**Fig. 4:**
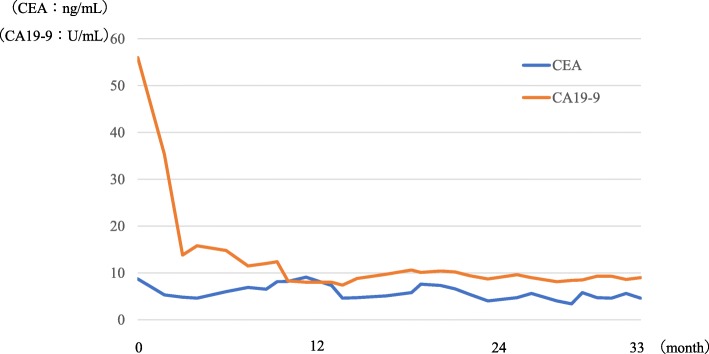
Transition of tumor markers. Serum CEA and CA19-9 levels returned to normal after the start of chemotherapy. CEA, carcinoembryonic antigen; CA19-9, carbohydrate antigen 19-9

After one course of the regimen, peritoneal lavage cytology returned negative for malignancy and remained negative throughout the treatment. CT showed gradually decreasing ascites (Fig. 3), whereas serum CEA and CA19-9 levels returned to normal (Fig. 4). Combined chemotherapy was continued without oral S-1 intake after the 15th course, whereas rest periods were extended from 1 to 2 weeks after the 34th course. Modifying the regimen and administration period allowed for the continuation of chemotherapy without major adverse effects with the patient remaining progression-free for 33 months with 36 chemotherapy cycles.

## Conclusions

Despite some progress in systemic chemotherapy [5–8], the median survival time (MST) of advanced/metastatic gastric cancer has still remained poor. PM of gastric cancer has been the most difficult pathology to detect and treat, with its prognosis being extremely poor. Moreover, given that patients with PM rarely have measurable lesions, only a few clinical trials have targeted patients with PM, whereas no established standard treatment has yet been available [9].

Recently, there has been growing concern regarding IP chemotherapy using PTX especially in Japan. PTX, which is hydrophobic and solubilized with Cremophor El® for clinical use, has a relatively large formulation size. Therefore, when administered intra-abdominally, PTX is not absorbed through vessels but is alternatively slowly absorbed from the peritoneum through the lymphatic system, which results in prolonged drug retention in the peritoneal cavity [9–11]. After IP infusion, PTX has shown substantially higher area under the curve ratios of intra-abdominal to systemic exposure compared with other hydrophilic drugs [12]. Given that exclusive IP administration of PTX had insufficient effect against the primary tumor and metastatic lymph node, IP combined with systemic chemotherapy, which was effective against advanced gastric cancer with PM, had been developed. With this therapeutic approach, the median survival reached 17.6–22.5 months, whereas 1 year survival exceeded 70% [9]. In a phase III trial comparing S-1 plus IP and IV PTX (IP arm) versus the standard Japanese regimen of S-1 plus cisplatin (SP arm) among patients having gastric cancer with PM, Ishigami et al. [2] failed to show better overall survival with the IP arm than with the SP arm (MST, 17.7 and 15.2 months, respectively; HR, 0.72; 95% CI, 0.49 to 1.04; *P* = .080) possibly due to baseline imbalance (i.e., the IP arm had significantly more patients with ascites) and deviations from protocol (i.e., some patients in the SP arm had received IP chemotherapy). Accordingly, sensitivity analysis adjusted for baseline ascites showed that the IP arm had a significantly better prognosis than the SP arm (adjusted HR, 0.59; 95% CI, 0.39 to 0.87; *P* = .008). Moreover, the 3-year overall survival rate was 21.9% (95% CI, 14.9 to 29.9%) in the IP arm and 6.0% (95% CI, 1.6 to 14.9%) in the SP arm. These results suggested that the Phoenix regimen with IP PTX is a promising treatment option for patients having gastric cancer with PM.

Although previous studies have investigated the efficacy of IP chemotherapy for gastric cancer with synchronous PM, only a few have investigated the effectiveness of IP chemotherapy for PR after curative surgery for gastric cancer. Another concern is that this is an early recurrence case after adjuvant S-1 monotherapy. Until the CLASSIC and JACCRO GC-07 trials demonstrated a survival benefit with the addition of oxaliplatin and docetaxel to oral fluoropyrimidine respectively [13, 14], S-1 monotherapy was a standard adjuvant treatment for patients with stage II and III gastric cancers based on the results of ACTS-GC trial [15]. However, approximately 30% of patients treated with S-1 adjuvant therapy exhibited a relapse. To make matters worse, reports suggested that early recurrence after S-1 adjuvant therapy is associated with a poor prognosis. Mitani reported that the median overall survival for patients with early recurrence after S-1 adjuvant therapy was 11.4 months [16]. In general, the treatment for patients with gastric cancer and PR after gastrectomy is systemic chemotherapy. As for treatment compliance of systemic chemotherapy, Andreyev revealed that patients with body weight loss (BWL) received significantly less chemotherapy and developed more toxicity [17]. However, BWL after gastrectomy is a common finding among patients with gastric cancer. Fein reported that BWL after gastrectomy was approximately 10–20% of the preoperative body weight [18]. Considering these factors, long-term treatment with systemic chemotherapy is difficult for patients with PR after gastrectomy. Regarding our case, partly because IP chemotherapy showed minimal systemic toxicity and partly because it offered an efficient and intensive regional therapy compared with systemic chemotherapy, he continued treatment for a long time despite showing recurrence after gastrectomy. Furthermore, regimens excluding S-1 also controlled his disease, suggesting that the IP administration of PTX was effective in this case.

To continue or discontinue treatment is another important concern. On an empirical basis, we continued IP chemotherapy as long as it remained effective with tolerable toxicity because some patients discontinued chemotherapy and developed early peritoneal recurrences. During the treatment, it is easy to perform a peritoneal lavage cytological examination through the intraperitoneal access port, and the results are used to determine the effects of IP chemotherapy. Recently, Ohzawa showed that the expression pattern of miRNAs in peritoneal exosomes reflects the tumor burden in the peritoneal cavity and suggested its potential as a useful biomarker in the treatment of PM [19]. Because patients with PM lack measurable lesions that are required to decide the therapy, future studies must discover reliable biomarkers reflecting tumor activity in the peritoneum.

The present case report details our experience with a patient suffering from gastric cancer with PR after curative surgery who had been successfully treated with IP and IV PTX combined with S-1 chemotherapy. Combination regimen, including IP chemotherapy, may therefore be a promising option for patients with PR after gastric cancer surgery.

## Data Availability

Not applicable.

## Abbreviations

PM: Peritoneal metastasis
IP: Intraperitoneal
PR: Peritoneal recurrence
IV: Intravenous
PTX: Paclitaxel
CEA: Carcinoembryonic antigen
CA19-9: Carbohydrate antigen 19-9
CT: Computed tomography
SL: Staging laparoscopy
MST: Median survival time
